# SLCO1B1*5 polymorphism (rs4149056) is associated with chemotherapy-induced amenorrhea in premenopausal women with breast cancer: a prospective cohort study

**DOI:** 10.1186/s12885-016-2373-3

**Published:** 2016-05-27

**Authors:** Toralf Reimer, Sarah Kempert, Bernd Gerber, Hans-Jürgen Thiesen, Steffi Hartmann, Dirk Koczan

**Affiliations:** Department of Obstetrics and Gynecology, University of Rostock, Klinikum Suedstadt, Suedring 81, Rostock, 18059 Germany; Institute of Immunology, University of Rostock, P.O.B. 100888, Rostock, 18055 Germany

**Keywords:** Breast cancer, Chemotherapy-induced amenorrhea, OpenArray genotyping, Single nucleotide polymorphism, SLCO1B1

## Abstract

**Background:**

Because inheritance is recognized as playing a role in age at menarche and natural menopause, the development of chemotherapy-induced amenorrhea (CIA) might depend on inherited genetic factors; however, studies that explore such a correlation are few and have received scant attention. Given the importance of this topic we conducted a comprehensive genotype study in young women (≤45 years) with early-stage breast cancer.

**Methods:**

Our approach tested the effect of variant polymorphisms in drug metabolism enzymes (DMEs) using a predesigned pharmacogenomics panel (TaqMan® OpenArray®, Life Technologies GmbH, Darmstadt, Germany) in premenopausal women (*n* = 50). Patients received contemporary chemotherapy; in all cases a cyclophosphamide-based regimen with a dose of at least 500 mg/m^2^ for six cycles. CIA was considered to be present in women with no resumption of menstrual bleeding within 12 months after completion of chemotherapy or goserelin.

**Results:**

Twenty-six patients (52 %) showed CIA during follow-up whereas 24 women (48 %) remained premenopausal. Of all the DMEs studied, only the SLCO1B1*5 (rs4149056) genotype was associated with the development of CIA (*P* = 0.017). Of the 26 patients who were homozygous for the T/T allele SLCO1B1*5, 18 (69.2 %) developed CIA compared with 8 (30.8 %) of the 22 patients who were heterozygous (C/T allele). The association of heterozygous SLCO1B1*5 allele (OR 0.038; 95%CI: 0.05–0.92) with a lower risk of developing CIA remained significant in a binary logistic regression analysis that include age, SLCO1B1*5 allele variants, and goserelin therapy.

**Conclusions:**

Patient age and SLCO1B1*5 allele variants predict the likelihood of young women with breast cancer developing CIA.

**Electronic supplementary material:**

The online version of this article (doi:10.1186/s12885-016-2373-3) contains supplementary material, which is available to authorized users.

## Background

In Germany, 10 % of all newly diagnosed breast cancers are detected in the age group under 45 years old [1]. Patients younger than 50 years of age, especially those with hormone-insensitive breast cancer, achieve significant benefit from adjuvant systemic chemotherapy in terms of prolonged disease-free and overall survival [2]. However, a considerable number of these young patients will suffer from chemotherapy-induced amenorrhea (CIA) thereafter, depending on age at diagnosis and type of chemotherapy used [3].

Premenopausal women in whom amenorrhea developed as a consequence of adjuvant breast cancer therapy had significantly better disease-free and overall survival than did women without amenorrhea, particularly when the tumor was estrogen receptor (ER)-positive [4]. Interestingly, the dose of the chemotherapy drug delivered was not a key factor in accounting for these differences [5]. Apart from its impact on survival and the loss of fertility, CIA due to premature ovarian failure leads to subjective and objective menopausal symptoms, which can negatively affect short- and long-term quality of life [6, 7].

Apart from its relationship with patient age at the time of therapy, there are no proven patient characteristics that are predictive of CIA [3, 8]. Pretreatment anti-Müllerian hormone (AMH), a biomarker of ovarian reserve, appears to be a potential predictor of CIA in women with early breast cancer [9]. However, further studies are needed in order to define the regimen-specific action of chemotherapy on AMH levels, the percentage of post-treatment recovery of AMH levels, and the relationship between menopausal status and AMH [10]. Because inheritance is recognized as playing a role in age at menarche and natural menopause [11, 12], the development of CIA might also depend on inherited genetic factors; however, studies that explore such a correlation are few and have received scant attention [13].

Previous pharmacogenetic investigations on CIA have focused on alkylating-based regimens. Cyclophosphamide is a pro-drug that requires activation by multiple cytochrome P450 (CYP450) enzymes [14]. The effect of cyclophosphamide on ovarian toxicity in patients with systemic lupus erythematosus has been found to be associated with the CYP2C19 genotype [15–17]. In another study women with CYP3A*1B variant genotype demonstrated a higher risk of ovarian failure after cyclophosphamide-based chemotherapy [18]. In conflict with these data, a pilot study investigating six CYP450 genotypes (*n* = 20) did not reveal a genetic effect on CIA in women with breast cancer receiving anthracycline- and cyclophosphamide-based therapy [19].

Solute carrier (SLC) transporters mediate the influx of cytotoxic drugs into cells. Organic anion-transporting polypeptides (OATPs) encoded by the solute carrier organic anion (SLCO) genes constitute an important transporter superfamily that mediates transmembrane transport of various clinical drugs and endogenous nutrients. Eleven human OATPs with different transport functions are expressed in various tissues. Many important clinical drugs have been identified as substrates of liver-specific OATP1B1 and OATP1B3 [20, 21].

SLCO1B1 encodes the organic anion-transporting polypeptide OATP1B1, which mediates the hepatic uptake of various drugs [22]. Previous studies have shown associations between genetic polymorphisms in SLCO genes and the uptake pharmacokinetics of their substrates. A common single-nucleotide variation (coding DNA c.521 T > C, protein p.V174A, rs4149056) in the SLCO1B1 gene decreases the transporting activity of OATP1B1, resulting in markedly increased plasma concentrations of many statins, for example [23].

Given the importance of this topic we planned a more comprehensive genotype study in a larger cohort of young women with early breast cancer. Our approach tested the effect of variant polymorphisms in drug metabolism enzymes (DMEs) using a predesigned pharmacogenomics panel in premenopausal women receiving contemporary chemotherapy.

## Methods

### Patients

A total of 50 premenopausal patients diagnosed with early-stage breast cancer between July 2001 and January 2011 were included in this prospective cohort analysis. All patients were aged ≤45 years at the time of diagnosis and were suitable to receive pre- or postoperative chemotherapy. In all cases patients received a cyclophosphamide-based regimen with a defined dose of at least 500 mg/m^2^ of body surface area for six cycles. Details of the protocols used in terms of administration of anthracyclines and/or taxanes are listed in Table 1.

**Table 1:** Summary of regimens used for pre- or postoperative chemotherapy in premenopausal women with early-stage breast cancer (*n* = 50)

Regimen	Patients n (%)	Cycles	Interval (weeks)	Dose per cycle		
				Cyclophosphamide	Anthracycline	Docetaxel
TAC	23 (46 %)	6	3	500 mg/m^2^	50 mg/m^2^ (A)	75 mg/m^2^
FEC	15 (30 %)	6	3	500 mg/m^2^	90 mg/m^2^ (E)	–
TC	8 (16 %)	6	3	600 mg/m^2^	–	75 mg/m^2^
EC	2 (4 %)	6	3	600 mg/m^2^	90 mg/m^2^ (E)	–
FAC	1 (2 %)	6	3	500 mg/m^2^	50 mg/m^2^ (A)	–
AC	1 (2 %)	6	3	600 mg/m^2^	60 mg/m^2^ (A)	–

*T* Taxotere® (docetaxel), *A* adriamycin (doxorubicin), *C* cyclophosphamide, *F* fluorouracil, *E* epirubicin

Only 30 % (*n* = 15) of all cases had hormone-insensitive breast cancer. Women with hormone receptor-positive disease (*n* = 35) received tamoxifen in a dosage of 20 mg daily for 5 years. Additionally, 50 % (*n* = 25) of included patients were treated with the gonadotrophin-releasing hormone (GnRH) agonist goserelin, 3.6 mg subcutaneously every 4 weeks, in two different clinical situations: 1) concurrent treatment with chemotherapy in hormone-insensitive breast cancer according to the ZOladex Rescue of Ovarian function (ZORO) study protocol (*n* = 4) [24]; or 2) concurrent and/or sequential goserelin application for a maximum of 2 years in hormone receptor-positive disease (*n* = 21).

### Clinical outcomes

At enrollment before chemotherapy, all women were premenopausal as defined by regularly occuring menstrual cycles. Follow-up assessment of menopausal status was performed 12 months after chemotherapy completion or 12 months from discontinuation of goserelin treatment. Normal ovarian function was defined as two consecutive menstrual periods within 21 to 35 days in a timeframe of 5 to 8 months after last administration of chemotherapy or goserelin. CIA was considered to be present in women with no resumption of menstrual bleeding within 12 months after completion of chemotherapy or goserelin. Plasma levels of follicle-stimulating hormone (FSH) and estradiol (E2) were measured during follow-up for serologic definition of menopausal status.

### DNA extraction and DME genotyping

Venous blood samples were taken prior to chemotherapy using PAXgene Blood DNA tubes (Qiagen, Hilden, Germany). DNA was purified according to the manufacturer’s instructions for the PAXgene Blood DNA kit (Qiagen) in a single-tube procedure. The homogenization of lysates was optimized by biopolymer-shredding system (QIAshredder, Qiagen). Extracted DNA was assessed with a micro-volume spectrophotometer (NanoDrop 8000, Fisher Scientific GmbH, Schwerte, Germany) and by analysis of housekeeping gene expression using conventional polymerase chain reaction (PCR). Additional DNA quantification was performed using Quant-iT™ DNA Assay kit (Invitrogen, Carlsbad, CA, USA), resulting in an optimal DNA concentration of 50 ng/μL.

The TaqMan® OpenArray® Pharmacogenomics (PGx) Panel (Life Technologies GmbH, Darmstadt, Germany) was used for genotyping analysis of DMEs and associated transport proteins. This OpenArray® genotyping plate includes assays for preformatted targets on a 192-well format (16 samples per panel; 3 sub-arrays per sample). Each TaqMan® DME Genotyping Assay contains two allele-specific probes and a primer pair to detect the specific SNP target. A total of 29 genes are covered across 164 unique assays (gene list available in Additional file 1: Table S1). Data were analyzed with OpenArray® SNP Genotyping Analysis software and then imported into TaqMan® Genotyper™ software.

### Statistical analysis

The Chi-squared test and Fisher’s exact test were used to compare categorical variables. The Mann-Whitney *U*-test was performed to compare continuous variables, and Spearman’s correlation coefficient is given, where appropriate. Binary logistic regression was performed to analyze the impact of factors predictive of CIA. Disease-free survival was analyzed using Kaplan-Meier method, with the log-rank test for comparison of subgroups. All analyses were conducted with the IBM SPSS Statistics Package, version 20 (IBM Deutschland GmbH, Ehningen, Germany). In all tests *P* < 0.05 was considered statistically significant, and all *P*-values cited were from two-tailed tests.

We performed post hoc analysis to estimate the statistical power of the described logistic regression analysis using the G*Power 3.1.9.2 tool [25]. Two-tailed analysis for prediction of CIA by the rs4149056 polymorphism with an α error of 0.05 revealed a statistical power of 70 % for our sample size.

## Results

Fifty subjects with complete data sets were included in this analysis. Twenty-six patients (52 %) showed CIA during follow-up whereas 24 women (48 %) remained premenopausal throughout the study. Among CIA subgroup (*n* = 26), serologic FSH and E2 assessment after completion of chemotherapy or goserelin confirmed peri- or postmenopausal status in the majority of cases: 15 postmenopausal (57.7 %), 8 perimenopausal (30.8 %), and 3 premenopausal (11.5 %). Table 2 shows the demographic and tumor characteristics for both subgroups (remained premenopausal vs. CIA). The only significant differences between the two groups were age (*P* = 0.001) and total duration of menstruation (*P* = 0.005). As expected, women who experienced CIA were significantly older than those who did not.

**Table 2:** Demographic and tumor characteristics of study population (Caucasian women, *n* = 50)

	Remained premenopausal (*n* = 24)	CIA (*n* = 26)	*P*-value
Mean age in years (± SD)	36.7 (4.8)	41.1 (4.0)	0.001
Mean BMI kg/m^2^ (± SD)	22.6 (2.9)	23.8 (3.8)	0.38
Smoking:*			1.0
No	20	20	
Yes	4	4	
Mean age in years at menarche (± SD)	13.1 (0.9)	12.8 (1.2)	0.44
Mean total duration of menstruation in years (± SD)	23.4 (5.1)	28.1 (4.8)	0.005
Mean parity (± SD)	1.4 (0.9)	1.7 (1.0)	0.49
Tumor stage:			0.21
pT1	14	18	
pT2	7	8	
pT3 & pT4	3	0	
Nodal stage:			0.82
pN0	11	11	
pN1	9	8	
pN2 & pN3	4	7	
Tumor grading:*			0.23
1	3	0	
2	10	12	
3	10	14	
Estrogen receptor status:			0.55
Positive	15	19	
Negative	9	7	
Progesterone receptor status:			0.77
Positive	16	16	
Negative	8	10	
HER2 status:			1.0
Positive	4	4	
Negative	20	22	
Tamoxifen therapy:			0.77
Yes	14	17	
No	10	9	
Goserelin therapy:			0.11
No	9	16	
Concurrent to chemotherapy	7	2	
Sequential to chemotherapy	8	8	
Taxan-based chemotherapy:			0.77
Yes	14	17	
No	10	9	

* denotes missing values

On analyzing the 164 assays of the PGx panel with respect to CIA, no statistics were computed for 89 assays (54.3 %) because results of these targets were constant, and 23 further assays (14 %) were excluded from the analysis because their frequencies were not in Hardy-Weinberg equilibrium. The remained 52 assays of interest were assessed for data quality, resulting in 34 finally evaluated assays (20.7 %) with complete and reproducible data sets (Additional file 2: Table S2, Additional file 3: Table S3).

Of all the DMEs studied, only the genotype of SLCO1B1*5 (rs4149056) was associated with the development of CIA (*P* = 0.017). Of the 26 patients who were homozygous for the SLCO1B1*5 T/T allele, 18 (69.2 %) developed CIA, compared with 8 (33.3 %) of the 24 patients who were either heterozygous or homozygous for the C allele (Table 3). SLCO1B1*5 genotype allele distribution as a function of chemotherapeutic regimen and patient age group is presented in Tables 4 and 5. The correlation between rs4149056 polymorphism and CIA was significant (Spearman r_s_ = 0.378, *P* = 0.007).

**Table 3:** SLCO1B1*5 genotype allele distribution as a function of resumption of menstrual bleeding (*n* = 50)

SLCO1B1*5 (rs4149056)	Remained premenopausal (*n* = 24)	CIA (*n* = 26)	*P*-value
T/T (*n* = 26)	8 (33.3 %)	18 (69.2 %)	0.017
C/T (*n* = 22)	14 (58.3 %)	8 (30.8 %)	
C/C (*n* = 2)	2 (8.4 %)	0

**Table 4:** SLCO1B1*5 genotype allele distribution as a function of chemotherapeutic regimen (*n* = 50)

rs4149056	TAC (*n* = 23)	FEC (*n* = 15)	TC (*n* = 8)	EC (*n* = 2)	FAC (*n* = 1)	AC (*n* = 1)	*P*-value
T/T	12	9	4	0	0	1	0.714
C/T	10	6	3	2	1	0	
C/C	1	0	1	0	0	0	

*T* Taxotere® (docetaxel), *A* adriamycin (doxorubicin), *C* cyclophosphamide, *F* fluorouracil, *E* epirubicin

**Table 5:** SLCO1B1*5 genotype allele distribution as a function of patient age group (*n* = 50)

rs4149056	<35 years (*n* = 10)	35–39 years (*n* = 13)	40–44 years (*n* = 20)	>44 years (*n* = 7)	*P*-value
T/T	5	6	11	4	0.703
C/T	5	7	8	2	
C/C	0	0	1	1	

Furthermore, the association of heterozygous SLCO1B1*5 allele with a lower risk of developing CIA remained significant on binary logistic regression analysis that include factors with a *P*-value < 0.2 in the univariate setting: age groups, SLCO1B1*5 allele variants, and goserelin therapy (Table 6). The protective effect of heterozygous SLCO1B1*5 allele regarding occurrence of CIA (odds ratio 0.22; 95 % CI 0.05–0.92) appears to be unrelated to patient age at initiation of chemotherapy. Regression analysis revealed that age (odds ratio for ≥40 years: 5.44; 95 % CI 1.29–22.9) contributed significantly to the model, indicating that age and SLCO1B1*5 allele variants predict the likelihood of developing CIA.

**Table 6:** Binary logistic regression for prediction of CIA: SLCO1B1*5 allele distribution and goserelin therapy as co-variates adjusted for age

Co-variates	*P*-value	Odds ratio	95 % CI
age (<40 vs. ≥40 years)	0.021	5.44	1.29–22.9
rs4149056 (T/T)	0.115	–	–
rs4149056 (C/C)	0.999	–	–
rs4149056 (C/T)	0.038	0.22	0.05–0.92
Goserelin therapy (no vs. yes)	0.17	0.49	0.18–1.35

Among this premenopausal study cohort, a recurrence of breast cancer was detected in 10 cases (20 %) during a median follow-up of 61 months (range 18–120 months). Disease-free survival was not related to occurrence of CIA (log-rank test, *P* = 0.526), SLCO1B1*5 genotype (*P* = 0.793), or age group (*P* = 0.985) using Kaplan-Meier method.

## Discussion

CIA is relevant because 25 % of women diagnosed with breast cancer are premenopausal, and previous studies suggest that CIA may impact breast cancer survival and patient’s quality of life due to premature ovarian failure with associated infertility and menopausal symptoms in women younger than 50 years.

Currently, little is known about the genetics of chemotherapy-induced ovarian failure and CIA. It is possible that the same genetic changes that are associated with premature menopause might also predict treatment-induced menopause [13]. A second category of candidate genes might be those that are implicated in drug metabolism. These include transporters involved in absorption and distribution, and in renal or hepatic elimination, and enzymes of drug metabolism that are usually present in the liver or gastrointestinal tract. SNPs in drug transporters, metabolizing enzymes and drug targets have the potential to affect therapeutic efficacy and toxicity [26, 27].

Our study results are a further example of the importance of variant polymorphisms predicting toxicities from chemotherapy in breast cancer patients. Using a pharmacogenomic approach permitting rapid scan of multiple markers, a significant association with CIA in Causasian women was found only for the SLCO1B1*5 allele distribution (rs4149056) (Table 6). In particular, patients who have the homozygous (T/T) genotype of the rs4149056 polymorphism showed a higher risk for developing CIA compared with patients who have the homozygous (C/C) or heterozygous (C/T) genotype.

In a genome-wide study the SEARCH Collaborative Group has identified the rs4149056 C allele in SLCO1B1 as being strongly associated with an increased risk of statin-induced myopathy [28]. No SNPs in any other region were clearly associated with myopathy. Several clinical studies in the past have investigated associations between rs4149056 SLCO1B1 genotypes and statin pharmacokinetics [22]. Although not all yielded significant results, the collective evidence indicates that statin blood concentrations are higher in people with the C allele [28].

Substrates for OATP1B1 include anticancer agents like methotrexate [29], gimatecan, SN-38 (the active metabolite of irinotecan), and pazopanib [23]. Certain SLCO1B1 variants associated with enhanced clearance of methotrexate increase the risk of gastrointestinal toxicity when this compound is used to treat children with acute lymphoblastic leukemia [30].

Endogenous substrates of SLCO1B1 include steroid hormone conjugates such as estradiol-17β-glucuronide and estrone-3-sulfate [31]. In a nested case-control study in the California Teachers Study cohort, only genetic variation in SLCO1B1 was associated with breast cancer risk. There was also an indication that estrogen-progestin therapy (EPT) may interact with SNPs in SLCO1B1; one variant (rs4149013) was significantly associated with breast cancer risk in EPT users [32].

Furthermore, given the frequent occurrence of overlapping substrate specificities – and the fact that functionally important coding-region SNPs in genes encoding members of some transporters families may not be common – it might be most fruitful to focus on combinations of coding-region SNPs in genes encoding transporters that work in parallel or in series [33]. In our data (Additional file 4: Table S4), alongside the described significant SLCO1B1*5 polymorphism in relation to CIA, there was a trend for a second SLC transporter genotype (SLC15A2: rs2257212, rs2293616) to be associated with menopausal status after completion of chemotherapy (*P* = 0.093).

In addition to the novel described potential genetic risk factor (SNP rs4149056) for CIA, increased age is unequivocally associated with higher rates of CIA. Older women, often defined as those at least 40 years old, are more sensitive to chemotherapy-related ovarian ablation [3]. This can be attributed to the decreasing number of active ovarian follicles with increasing age [34]. Taking the cut-off of 40 years, we found significantly different incidences of CIA in the age group <40 years (30.4 %) compared with the subgroup ≥40 years (70.4 %) when using contemporary chemotherapeutic regimens. Recently, the pharmacogenetic pilot study by Wessels et al. [19] including common variant alleles for CYP450 enzymes found no differences in genotype frequencies but, as expected, women who experienced CIA were significantly older than those who did not (*P* = 0.009). The risk of CIA in young patients with breast cancer (≤47 years) does not appear to be related to BRCA1 or BRCA2 mutation status [35]. Again, age at treatment and use of tamoxifen were important predictors of CIA in women who carry a BRCA1 or BRCA2 mutation.

The major limitations of the study are the limited case number (*n* = 50) leading to a reduced statistical power of 70 %, and the inclusion of patients with goserelin treatment. Previous studies have suggested that temporary ovarian suppression with a GnRH analogue may preserve ovarian function both in humans and animal models [36]. However, the clinical data are conflicting. For example, the ZORO study randomised 60 ER-negative patients with breast cancer to anthracycline- and taxane-based chemotherapy alone or in combination with goserelin and found that there was no statistical difference in the resumption of ovarian function between the two groups [24]. This finding is confirmed in recent publications of other prospective trials [37, 38]. Currently, following publication of the Prevention of Early Menopause Study (POEMS) results [39, 40], the use of GnRH agonists during adjuvant chemotherapy outside clinical trials is being re-evaluated. Our data showed a trend for preservation of menstrual bleeding in patients receiving goserelin treatment concurrently with chemotherapy, but the difference was not statistically significant (Table 2). Administration of goserelin sequentially to chemotherapy as endocrine therapy in ER-positive disease had no impact on the occurrence of CIA.

Other methodologic concerns include inconsistencies in defining CIA and variability in the use of chemotherapeutic agents and tamoxifen in heterogeneous study population, depending on hormone-receptor status. However, our data do not support the hypothesis or previous findings that taxane-based chemotherapy or tamoxifen may have an impact on CIA (Table 2). For example, Najafi et al. [41] have reported that the type of chemotherapy is an important risk factor for CIA and that taxane-based regimens induced more CIA than other regimens. Non-cyclophosphamide treated controls or breast cancer patients without chemotherapy were not included in our study.

The lack of a consistent definition across breast cancer literature complicates incidence comparisons between different regimens and studies. The definition of CIA ranges from one missed period within 1 year to continuous cessation of menses for more than 1 year [3]. In summary, irreversible amenorrhea lasting for several (>12) months following chemotherapy and an FSH level of ≥30 IU/L in the presence of a negative pregnancy test seems to be an appropriate characterization of chemotherapy-induced ovarian failure [42]. We used strict criteria to define CIA as no resumption of menstrual bleeding within 12 months after completion of chemotherapy or goserelin. However, cessation of menses is not synonymous with true ovarian failure because estrogen levels can remain in the premenopausal range even where CIA persists for 1 year or longer (as in 10 % of our cases with CIA).

## Conclusions

This pharmacogenomics study revealed a genetic effect on CIA in Caucasian women with early breast cancer receiving standard contemporary chemotherapy. The impact on CIA is due to genotype distribution of the SLC drug transporter gene SLCO1B1*5. A prospective validation of this finding is required in a larger cohort of premenopausal women using clearly predefined inclusion criteria (e.g., no treatment with goserelin). These data illustrate the potential power for predicting CIA based on a more profound understanding of inherited genetic factors.

## Additional files

Additional file 1: Table S1. List of gene targets in the TaqMan® OpenArray® Pharmacogenomics (PGx) Panel (*source: www.PharmaADME.org). Phase I and II metabolism enzymes, responsible for the modification of functional groups and the conjugation with endogenous moieties respectively; transporters, responsible for the uptake and excretion of drugs in and out of cells. (DOCX 13 kb) Additional file 2: Table S2. List of finally evaluated 34 assays using TaqMan® OpenArray® PGx Panel. (DOCX 14 kb) Additional file 3: Table S3. List of non-evaluated 130 assays using TaqMan® OpenArray® PGx Panel due to the following reasons: 1…target revealed a constant of 100 % for one allele polymorphism (*n* = 89), 2…detected frequencies were not in Hardy-Weinberg equilibrium (*n* = 23), 3…incomplete or non-reproducible data sets (*n* = 17). *…assay C_11711720C_30 and assay C_11711720D_40 focused on the identical polymorphism (rs2032582). (DOCX 18 kb) Additional file 4: Table S4. List of 33 computed, non-significant assays of interest with respect to menstrual bleeding after end of chemotherapy. (DOCX 21 kb)
