# Supplementary material for: SLCO1B1*5 polymorphism (rs4149056) is associated with chemotherapy-induced amenorrhea in premenopausal women with breast cancer: a prospective cohort study
Source: BMC Cancer. 2016 May 27;16:337. doi: 10.1186/s12885-016-2373-3 (PMC4884353; doi:10.1186/s12885-016-2373-3)
Supplement: Additional file 1: Table S1. — List of gene targets in the TaqMan® OpenArray® Pharmacogenomics (PGx) Panel (*source: www.PharmaADME.org). Phase I and II metabolism enzymes, responsible for the modification of functional groups and the conjugation with endogenous moieties respectively; transporters, responsible for the uptake and excretion of drugs in and out of cells. (DOCX 13 kb) [file 12885_2016_2373_MOESM1_ESM.docx]

Additional file 1: Table S1: List of gene targets in the TaqMan® OpenArray® Pharmacogenomics (PGx) Panel (*source: www.PharmaADME.org). Phase I and II metabolism enzymes, responsible for the modification of functional groups and the conjugation with endogenous moieties respectively; transporters, responsible for the uptake and excretion of drugs in and out of cells.

| PharmaADME class* | Number of genes | Human gene symbols |
| --- | --- | --- |
| Phase I | 12 | CYP1A1, CYP1A2, CYP2A6, CYP2B6, CYP2C8, CYP2C9, CYP2C19, CYP2D6, CYP2E1, CYP3A4, CYP3A5, DPYD |
| Phase II | 6 | GSTP1, NAT1, NAT2, UGT1A1, UGT2B7, UGT2B15 |
| Transporter | 11 | ABCB1, ABCC2, ABCG2, SLC15A2, SLC22A1, SLC22A2, SLC22A6, SLCO1B1, SLCO1B3, SLCO2B1, TPMT |
