# Supplementary material for: SLCO1B1*5 polymorphism (rs4149056) is associated with chemotherapy-induced amenorrhea in premenopausal women with breast cancer: a prospective cohort study
Source: BMC Cancer. 2016 May 27;16:337. doi: 10.1186/s12885-016-2373-3 (PMC4884353; doi:10.1186/s12885-016-2373-3)
Supplement: Additional file 2: Table S2. — List of finally evaluated 34 assays using TaqMan® OpenArray® PGx Panel. (DOCX 14 kb) [file 12885_2016_2373_MOESM2_ESM.docx]

Additional file 2: Table S2: List of finally evaluated 34 assays using TaqMan® OpenArray® PGx Panel.

| Gene | Star nomenclature | Nucleotide change | Amino acid change | RS # |
| --- | --- | --- | --- | --- |
| ABCB1 |  | 3435C>T |  | rs1045642 |
| ABCB1 |  | T1236T>C |  | rs1128503 |
| ABCB1 |  | 2677T>G,A |  | rs2032582 |
| ABCC2 |  |  | V417I | rs2273697 |
| ABCC2 |  |  | I1324I | rs3740066 |
| ABCG2 |  |  | 421C>A | rs2231142 |
| CYP2C8 | *3 |  | K399R | rs10509681 |
| CYP2C8 | *3 |  | R139K | rs11572080 |
| CYP2C9 | *2 |  |  | rs1799853 |
| CYP2C9 | *3 |  |  | rs1057910 |
| CYP2C19 | *2 | 80161A>G |  | rs4244285 |
| CYP2C19 | *17 | -806C>T |  | rs12248560 |
| CYP2D6 | *2a |  |  | rs1080985 |
| CYP2D6 | *10 | 100C>T |  | rs1065852 |
| CYP3A5 | *3 | 6986A>G |  | rs776746 |
| DPYD | *9A |  | C29R | rs1801265 |
| GSTP1 | *A_*B_*C_*D |  | V105I | rs1695 |
| NAT2 | *5 | 341T>C |  | rs1801280 |
| NAT2 | *6 |  |  | rs1799930 |
| NAT2 | *11 | 481C>T |  | rs1799929 |
| NAT2 | *12 | 803A>G |  | rs1208 |
| SLC15A2 |  |  | L350F | rs2257212 |
| SLC15A2 |  |  | A284A | rs2293616 |
| SLC22A1 |  |  | M408V | rs628031 |
| SLC22A1 |  |  |  | rs72552763 |
| SLC22A2 | *4 |  | S270A | rs316019 |
| SLCO1B1 | *1B |  | N130D | rs2306283 |
| SLCO1B1 | *5 | 521T>C | V174A | rs4149056 |
| SLCO1B3 |  | 334T>G | A112S | rs4149117 |
| SLCO1B3 |  | 699G>A | I233M | rs7311358 |
| UGT1A1 | *60 |  |  | rs4124874 |
| UGT2B7 |  |  |  | rs7668258 |
| UGT2B7 |  |  |  | rs7662029 |
| UGT2B15 | *2 |  | D85Y | rs1902023 |
