# Supplementary material for: SLCO1B1*5 polymorphism (rs4149056) is associated with chemotherapy-induced amenorrhea in premenopausal women with breast cancer: a prospective cohort study
Source: BMC Cancer. 2016 May 27;16:337. doi: 10.1186/s12885-016-2373-3 (PMC4884353; doi:10.1186/s12885-016-2373-3)
Supplement: Additional file 3: Table S3. — List of non-evaluated 130 assays using TaqMan® OpenArray® PGx Panel due to the following reasons: 1…target revealed a constant of 100 % for one allele polymorphism (n = 89), 2…detected frequencies were not in Hardy-Weinberg equilibrium (n = 23), 3…incomplete or non-reproducible data sets (n = 17). *…assay C_11711720C_30 and assay C_11711720D_40 focused on the identical polymorphism (rs2032582). (DOCX 18 kb) [file 12885_2016_2373_MOESM3_ESM.docx]

Additional file 3: Table S3: List of non-evaluated 130 assays using TaqMan® OpenArray® PGx Panel due to the following reasons:

1...target revealed a constant of 100% for one allele polymorphism (n=89),

2...detected frequencies were not in Hardy-Weinberg equilibrium (n=23),

3...incomplete or non-reproducible data sets (n=17).

*...assay C_11711720C_30 and assay C_11711720D_40 focused on the identical polymorphism (rs2032582)

| Gene | Star nomenclature | Nucleotide change | RS # | No evaluation due to |
| --- | --- | --- | --- | --- |
| ABCB1 |  | A/G | rs3213619 | 1 |
| ABCC2 |  | C/T | rs56199535 | 1 |
| ABCC2 |  | C/T | rs56220353 | 1 |
| ABCC2 |  | A/G | rs56296335 | 1 |
| ABCG2 |  | G/A | rs72552713 | 1 |
| CYP1A1 | *3 | A/G | rs1800031 | 1 |
| CYP1A1 | *5 | G/T | rs41279188 | 1 |
| CYP1A1 | *5 | G/A | rs41279188 | 1 |
| CYP1A1 | *6 | C/T | rs56313657 | 1 |
| CYP1A1 | *7 | A/- | rs72547510 | 1 |
| CYP1A1 | *8 | A/T | rs72547509 | 1 |
| CYP1A2 |  | A/G | rs56107638 | 1 |
| CYP1A2 | *1K | C/T | rs12720461 | 1 |
| CYP2A6 | *5 | G/T | rs66497551 | 1 |
| CYP2A6 | *6 | C/T | rs4986891 | 1 |
| CYP2A6 | *7 | C/T | rs72547591 | 1 |
| CYP2A6 | *13 | A/C | rs28399433 | 1 |
| CYP2A6 | *17 | C/T | rs28399454 | 1 |
| CYP2A6 | *20 | TT/- | rs- | 1 |
| CYP2A13 |  | C/T | rs- | 1 |
| CYP2B6 | *16 | C/T | rs28399499 | 1 |
| CYP2C8 | *2 | A/T | rs11572103 | 1 |
| CYP2C8 | *5 | T/- | rs72558196 | 1 |
| CYP2C8 | *7 | G/C | rs72558195 | 1 |
| CYP2C8 | *7 | G/A | rs72558195 | 1 |
| CYP2C9 | *5 | C/G | rs28371686 | 1 |
| CYP2C9 | *6 | -/A | rs- | 1 |
| CYP2C9 | *9 | A/G | rs2256871 | 1 |
| CYP2C9 | *10 | G/A | rs9332130 | 1 |
| CYP2C9 | *12 | C/T | rs9332239 | 1 |
| CYP2C9 | *13 | C/T | rs74052158 | 1 |
| CYP2C9 | *15 | A/C | rs72558190 | 1 |
| CYP2C9 | *25 | - | rs- | 1 |
| CYP2C9 | *27 | T/G | rs7900194 | 1 |
| CYP2C9 | *27 | A/G | rs7900194 | 1 |
| CYP2C19 | *3 | A/G | rs4986893 | 1 |
| CYP2C19 | *4 | A/G | rs28399504 | 1 |
| CYP2C19 | *5A | C/T | rs56337013 | 1 |
| CYP2C19 | *7 | A/T | rs72558186 | 1 |
| CYP2C19 | *8 | C/T | rs41291556 | 1 |
| CYP2C19 | *12 | A/C | rs55640102 | 1 |
| CYP2D6 | *7 | G/T | rs5030867 | 1 |
| CYP2D6 | *11 | C/G | rs5030863 | 1 |
| CYP2D6 | *12 | C/T | rs5030862 | 1 |
| CYP2D6 | *14A | A/C | rs5030865 | 1 |
| CYP2D6 | *14A | T/C | rs5030865 | 1 |
| CYP2D6 | *17 | G/A | rs28371706 | 1 |
| CYP2D6 | *18 | - | rs- | 1 |
| CYP2D6 | *19 | -/AGTT | rs- | 1 |
| CYP2D6 | *20 | C/- | rs72549354 | 1 |
| CYP2D6 | *30 | - | rs- | 1 |
| CYP2D6 | *38 | AGTC/- | rs72549351 | 1 |
| CYP2D6 | *42 | AC/- | rs72549346 | 1 |
| CYP2D6 | *44 | G/C | rs72549349 | 1 |
| CYP2D6 | *56A | A/G | rs- | 1 |
| CYP2E1 | *2 | G/A | rs72559710 | 1 |
| CYP3A4 | *2 | A/G | rs55785340 | 1 |
| CYP3A4 | *6 | T/- | rs4646438 | 1 |
| CYP3A5 | *3G | A/G | rs55965422 | 1 |
| CYP3A5 | *7 | A/- | rs41303343 | 1 |
| CYP3A5 | *10 | A/G | rs41279854 | 1 |
| DPYD |  | A/C | rs1801268 | 1 |
| DPYD |  | -/ATGA | rs- | 1 |
| DPYD |  | A/G | rs1801266 | 1 |
| GSTP1 |  | C/T | rs1138272 | 1 |
| NAT1 | *5 | A/G | rs55793712 | 1 |
| NAT1 | *15 | C/T | rs5030839 | 1 |
| NAT1 | *19 | C/T | rs56318881 | 1 |
| NAT2 | *14A | A/G | rs1801279 | 1 |
| SLCO1B1 |  | C/T | rs56101265 | 1 |
| SLCO1B1 |  | C/T | rs56061388 | 1 |
| SLCO1B1 |  | A/G | rs7255974 | 1 |
| SLCO1B1 |  | C/T | rs55901008 | 1 |
| SLCO1B1 |  | C/G | rs59502379 | 1 |
| SLCO1B1 |  | A/G | rs56199088 | 1 |
| SLCO1B1 |  | G/A | rs55737008 | 1 |
| SLC22A1 |  | G/T | rs36103319 | 1 |
| SLC22A2 |  | A/G | rs8177516 | 1 |
| SLC22A2 |  | G/A | rs8177504 | 1 |
| SLC22A2 |  | G/T | rs8177517 | 1 |
| SLC22A2 |  | C/T | rs8177507 | 1 |
| SLC22A6 |  | C/T | rs11568626 | 1 |
| TPMT |  | C/T | rs1800584 | 1 |
| TPMT |  | C/G | rs1800462 | 1 |
| TPMT |  | C/T | rs56161402 | 1 |
| UGT1A1 | *6 | A/G | rs4148323 | 1 |
| UGT1A1 | *7 | G/T | rs34993780 | 1 |
| UGT1A1 | *27 | A/C | rs35350960 | 1 |
| UGT1A1 | *29 | C/G | rs55750087 | 1 |
| CYP1A1 | *2B | C/T | rs1048943 | 2 |
| CYP1A1 | *4 | T/G | rs1799814 | 2 |
| CYP2A6 | *2 | T/A | rs1801272 | 2 |
| CYP2B6 | *13A | A/G | rs12721655 | 2 |
| CYP2B6 | *28 | C/T | rs34097093 | 2 |
| CYP2C8 | *4 | C/G | rs1058930 | 2 |
| CYP2C9 | *11A | T/C | rs28371685 | 2 |
| CYP2C19 | *6 | A/G | rs72552267 | 2 |
| CYP2D6 | *3A | T/- | rs35742686 | 2 |
| CYP2D6 | *6A | A/- | rs5030655 | 2 |
| DPYD |  | C/T | rs3918290 | 2 |
| DPYD |  | C/T | rs1801267 | 2 |
| NAT1 | *11A | C/T | rs4986988 | 2 |
| NAT1 | *14A | A/G | rs4986782 | 2 |
| NAT1 | *17 | C/T | rs56379106 | 2 |
| NAT1 | *22 | A/T | rs56172717 | 2 |
| NAT2 | *7A | A/G | rs1799931 | 2 |
| SLC22A1 |  | A/G | rs3405908 | 2 |
| SLC22A1 |  | C/T | rs2282143 | 2 |
| SLC22A1 |  | C/T | rs55918055 | 2 |
| SLCO2B1 |  | C/T | rs2306168 | 2 |
| TPMT |  | T/C | rs1800460 | 2 |
| TPMT |  | C/T | rs1142345 | 2 |
| ABCC2 |  | C/T | rs717620 | 3 |
| CYP1A2 | *1C | G/A | rs2069514 | 3 |
| CYP1A2 | *1Q | C/A | rs762551 | 3 |
| CYP2B6 | *13A | G/T | rs3745274 | 3 |
| CYP2D6 | *2M | C/T | rs28371725 | 3 |
| CYP2D6 | *4A | C/T | rs3892097 | 3 |
| CYP2D6 | *9 | TCT/- | rs72549350 | 3 |
| CYP2D6 | *15 | -/A | rs- | 3 |
| CYP3A5 | *6 | C/T | rs10264272 | 3 |
| GSTM1 |  | C/G | rs74837985 | 3 |
| NAT2 | *12B | C/T | rs1041983 | 3 |
| NAT2 | *19 | C/T | rs1805158 | 3 |
| SLC15A2 |  | A/G | rs1143672 | 3 |
| SLC15A2 |  | C/T | rs1143671 | 3 |
| SLC22A1 |  | C/G | rs4646278 | 3 |
| SLC22A1 |  | C/T | rs4646277 | 3 |
| SLC22A1 |  | C/T | rs12208357 | 3 |
