# Supplementary material for: SLCO1B1*5 polymorphism (rs4149056) is associated with chemotherapy-induced amenorrhea in premenopausal women with breast cancer: a prospective cohort study
Source: BMC Cancer. 2016 May 27;16:337. doi: 10.1186/s12885-016-2373-3 (PMC4884353; doi:10.1186/s12885-016-2373-3)
Supplement: Additional file 4: Table S4. — List of 33 computed, non-significant assays of interest with respect to menstrual bleeding after end of chemotherapy. (DOCX 21 kb) [file 12885_2016_2373_MOESM4_ESM.docx]

Additional file 4: Table S4: List of 33 computed, non-significant assays of interest with respect to menstrual bleeding after end of chemotherapy.

| ABCB1 (rs1045642) | Remained premenopausal  (n=24) | CIA  (n=26) | P-value |
| --- | --- | --- | --- |
| G/G (n=13) | 6 (25.0%) | 7 (26.9%) | 0.978 |
| A/G (n=21) | 10 (41.7%) | 11 (42.3%) |  |
| A/A (n=16) | 8 (33.3%) | 8 (30.8%) |  |

| ABCB1 (rs1128503) | Remained premenopausal  (n=24) | CIA  (n=26) | P-value |
| --- | --- | --- | --- |
| G/G (n=17) | 5 (20.8%) | 12 (46.2%) | 0.137 |
| A/G (n=24) | 13 (54.2%) | 11 (42.3%) |  |
| A/A (n=9) | 6 (25.0%) | 3 (11.5%) |  |

| ABCB1 (rs2032582)  (n=1 undetected) | Remained premenopausal  (n=24) | CIA  (n=26) | P-value |
| --- | --- | --- | --- |
| T/T (n=9) | 5 (20.8%) | 4 (15.4%) | 0.466 |
| C/T (n=23) | 12 (50.0%) | 11 (42.3%) |  |
| C/C (n=17) | 6 (25.0%) | 11 (42.3%) |  |

| ABCC2 (rs2273697) | Remained premenopausal  (n=24) | CIA  (n=26) | P-value |
| --- | --- | --- | --- |
| G/G (n=39) | 19 (79.2%) | 20 (76.9%) | 0.848 |
| A/G (n=11) | 5 (20.8%) | 6 (23.1%) |  |
| A/A (n=0) | 0 | 0 |  |

| ABCC2 (rs3740066) | Remained premenopausal  (n=24) | CIA  (n=26) | P-value |
| --- | --- | --- | --- |
| T/T (n=9) | 4 (16.7%) | 5 (19.2%) | 0.532 |
| C/T (n=21) | 12 (50.0%) | 9 (34.6%) |  |
| C/C (n=20) | 8 (33.3%) | 12 (46.2%) |  |

| ABCG2 (rs2231142) | Remained premenopausal  (n=24) | CIA  (n=26) | P-value |
| --- | --- | --- | --- |
| T/T (n=1) | 1 (4.2%) | 0 | 0.192 |
| G/T (n=11) | 3 (12.5%) | 8 (30.8%) |  |
| G/G (n=38) | 20 (83.3%) | 18 (69.2%) |  |

| CYP2C8*3 (rs10509681) | Remained premenopausal  (n=24) | CIA  (n=26) | P-value |
| --- | --- | --- | --- |
| T/T (n=43) | 21 (87.5%) | 22 (84.6%) | 0.769 |
| C/T (n=7) | 3 (12.5%) | 4 (15.4%) |  |
| C/C (n=0) | 0 | 0 |  |

| CYP2C8*3 (rs11572080) | Remained premenopausal  (n=24) | CIA  (n=26) | P-value |
| --- | --- | --- | --- |
| T/T (n=0) | 0 | 0 | 0.769 |
| C/T (n=7) | 3 (12.5%) | 4 (15.4%) |  |
| C/C (n=43) | 21 (87.5%) | 22 (84.6%) |  |

| CYP2C9*2 (rs1799853) | Remained premenopausal  (n=24) | CIA  (n=26) | P-value |
| --- | --- | --- | --- |
| T/T (n=1) | 0 | 1 (3.8%) | 0.624 |
| C/T (n=6) | 3 (12.5%) | 3 (11.5%) |  |
| C/C (n=43) | 21 (87.5%) | 22 (84.6%) |  |

| CYP2C9*3 (rs1057910) | Remained premenopausal  (n=24) | CIA  (n=26) | P-value |
| --- | --- | --- | --- |
| A/A (n=43) | 20 (83.3%) | 23 (88.5%) | 0.602 |
| C/A (n=7) | 4 (16.7%) | 3 (11.5%) |  |
| C/C (n=0) | 0 | 0 |  |

| CYP2C19*2 (rs4244285) | Remained premenopausal  (n=24) | CIA  (n=26) | P-value |
| --- | --- | --- | --- |
| G/G (n=40) | 19 (79.2%) | 21 (80.8%) | 0.887 |
| A/G (n=10) | 5 (20.8%) | 5 (19.2%) |  |
| A/A (n=0) | 0 | 0 |  |

| CYP2C19*17 (rs12248560) | Remained premenopausal  (n=24) | CIA  (n=26) | P-value |
| --- | --- | --- | --- |
| T/T (n=2) | 1 (4.2%) | 1 (3.8%) | 0.881 |
| C/T (n=12) | 5 (20.8%) | 7 (26.9%) |  |
| C/C (n=36) | 18 (75.0%) | 18 (69.2%) |  |

| CYP2D6*2a (rs1080985) | Remained premenopausal  (n=24) | CIA  (n=26) | P-value |
| --- | --- | --- | --- |
| C/C (n=4) | 2 (8.4%) | 2 (7.7%) | 0.552 |
| G/C (n=14) | 5 (20.8%) | 9 (34.6%) |  |
| G/G (n=32) | 17 (70.8%) | 15 (57.7%) |  |

| CYP2D6*10 (rs1065852) | Remained premenopausal  (n=24) | CIA  (n=26) | P-value |
| --- | --- | --- | --- |
| G/G (n=28) | 14 (58.4%) | 14 (53.8%) | 0.853 |
| A/G (n=17) | 7 (29.3%) | 10 (38.4%) |  |
| A/A (n=2) | 1 (4.3%) | 1 (3.8%) |  |

| CYP3A5*3 (rs776746) | Remained premenopausal  (n=24) | CIA  (n=26) | P-value |
| --- | --- | --- | --- |
| C/C (n=44) | 22 (91.7%) | 22 (84.7%) | 0.571 |
| T/C (n=5) | 2 (8.3%) | 3 (11.5%) |  |
| T/T (n=1) | 0 | 1 (3.8%) |  |

| DPYD*9A (rs1801265) | Remained premenopausal  (n=24) | CIA  (n=26) | P-value |
| --- | --- | --- | --- |
| G/G (n=2) | 1 (4.2%) | 1 (3.8%) | 0.839 |
| A/G (n=23) | 10 (41.6%) | 13 (50.0%) |  |
| A/A (n=25) | 13 (54.2%) | 12 (46.2%) |  |

| GSTP1*A_*B_*C_*D  (rs1695; n=1 undetected) | Remained premenopausal  (n=24) | CIA  (n=26) | P-value |
| --- | --- | --- | --- |
| G/G (n=3) | 1 (4.2%) | 2 (7.7%) | 0.703 |
| A/G (n=20) | 10 (41.8%) | 10 (38.5%) |  |
| A/A (n=26) | 12 (50.0%) | 14 (53.8%) |  |

| NAT2*5 (rs1801280) | Remained premenopausal  (n=24) | CIA  (n=26) | P-value |
| --- | --- | --- | --- |
| T/T (n=13) | 6 (25.0%) | 7 (26.9%) | 0.886 |
| C/T (n=26) | 12 (50.0%) | 14 (53.8%) |  |
| C/C (n=11) | 6 (25.0%) | 5 (19.3%) |  |

| NAT2*6 (rs1799930) | Remained premenopausal  (n=24) | CIA  (n=26) | P-value |
| --- | --- | --- | --- |
| G/G (n=28) | 16 (66.6%) | 12 (46.2%) | 0.317 |
| A/G (n=20) | 7 (29.2%) | 13 (50.0%) |  |
| A/A (n=2) | 1 (4.2%) | 1 (3.8%) |  |

| NAT2*11 (rs1799929) | Remained premenopausal  (n=24) | CIA  (n=26) | P-value |
| --- | --- | --- | --- |
| T/T (n=9) | 5 (20.8%) | 4 (15.4%) | 0.838 |
| C/T (n=27) | 13 (54.2%) | 14 (53.8%) |  |
| C/C (n=14) | 6 (25.0%) | 8 (30.8%) |  |

| NAT2*12 (rs1208) | Remained premenopausal  (n=24) | CIA  (n=26) | P-value |
| --- | --- | --- | --- |
| G/G (n=12) | 7 (29.2%) | 5 (19.2%) | 0.702 |
| A/G (n=24) | 11 (45.8%) | 13 (50.0%) |  |
| A/A (n=14) | 6 (25.0%) | 8 (30.8%) |  |

| SLC15A2 (rs2257212) | Remained premenopausal  (n=24) | CIA  (n=26) | P-value |
| --- | --- | --- | --- |
| T/T (n=13) | 3 (12.5%) | 10 (38.5%) | 0.093 |
| C/T (n=21) | 11 (45.8%) | 10 (38.5%) |  |
| C/C (n=16) | 10 (41.7%) | 6 (23.0%) |  |

| SLC15A2 (rs2293616) | Remained premenopausal  (n=24) | CIA  (n=26) | P-value |
| --- | --- | --- | --- |
| G/G (n=16) | 10 (41.7%) | 6 (23.0%) | 0.093 |
| A/G (n=21) | 11 (45.8%) | 10 (38.5%) |  |
| A/A (n=13) | 3 (12.5%) | 10 (38.5%) |  |

| SLC22A1 (rs628031) | Remained premenopausal  (n=24) | CIA  (n=26) | P-value |
| --- | --- | --- | --- |
| A/A (n=8) | 5 (20.8%) | 3 (11.5%) | 0.458 |
| G/A (n=23) | 9 (37.5%) | 14 (53.9%) |  |
| G/G (n=19) | 10 (41.7%) | 9 (34.6%) |  |

| SLC22A1 (rs72552763) | Remained premenopausal  (n=24) | CIA  (n=26) | P-value |
| --- | --- | --- | --- |
| GAT/GAT (n=33) | 14 (58.3%) | 19 (73.1%) | 0.253 |
| -/GAT (n=15) | 8 (33.3%) | 7 (26.9%) |  |
| -/- (n=2) | 2 (8.4%) | 0 |  |

| SLC22A2*4 (rs316019) | Remained premenopausal  (n=24) | CIA  (n=26) | P-value |
| --- | --- | --- | --- |
| C/C (n=41) | 19 (79.2%) | 22 (84.6%) | 0.868 |
| A/C (n=7) | 4 (16.7%) | 3 (11.5%) |  |
| A/A (n=2) | 1 (4.1%) | 1 (3.9%) |  |

| SLCO1B1*1B (rs2306283) | Remained premenopausal  (n=24) | CIA  (n=26) | P-value |
| --- | --- | --- | --- |
| G/G (n=11) | 7 (29.2%) | 4 (15.4%) | 0.348 |
| A/G (n=27) | 13 (54.2%) | 14 (53.8%) |  |
| A/A (n=12) | 4 (16.6%) | 8 (30.8%) |  |

| SLCO1B3 (rs4149117) | Remained premenopausal  (n=24) | CIA  (n=26) | P-value |
| --- | --- | --- | --- |
| T/T (n=2) | 0 | 2 (7.7%) | 0.366 |
| G/T (n=19) | 9 (37.5%) | 10 (38.5%) |  |
| G/G (n=29) | 15 (62.5%) | 14 (53.8%) |  |

| SLCO1B3 (rs7311358) | Remained premenopausal  (n=24) | CIA  (n=26) | P-value |
| --- | --- | --- | --- |
| G/G (n=2) | 0 | 2 (7.7%) | 0.366 |
| A/G (n=19) | 9 (37.5%) | 10 (38.5%) |  |
| A/A (n=29) | 15 (62.5%) | 14 (53.8%) |  |

| UGT1A1*60 (rs4124874) | Remained premenopausal  (n=24) | CIA  (n=26) | P-value |
| --- | --- | --- | --- |
| T/T (n=13) | 6 (25.0%) | 7 (26.9%) | 0.131 |
| G/T (n=28) | 11 (45.8%) | 17 (65.4%) |  |
| G/G (n=9) | 7 (29.2%) | 2 (7.7%) |  |

| UGT2B7 (rs7668258) | Remained premenopausal  (n=24) | CIA  (n=26) | P-value |
| --- | --- | --- | --- |
| T/T (n=13) | 7 (29.2%) | 6 (23.1%) | 0.805 |
| C/T (n=27) | 13 (54.2%) | 14 (53.8%) |  |
| C/C (n=10) | 4 (16.6%) | 6 (23.1%) |  |

| UGT2B7 (rs7662029) | Remained premenopausal  (n=24) | CIA  (n=26) | P-value |
| --- | --- | --- | --- |
| G/G (n=10) | 4 (16.6%) | 6 (23.1%) | 0.805 |
| A/G (n=27) | 13 (54.2%) | 14 (53.8%) |  |
| A/A (n=13) | 7 (29.2%) | 6 (23.1%) |  |

| UGT2B15*2 (rs1902023) | Remained premenopausal  (n=24) | CIA  (n=26) | P-value |
| --- | --- | --- | --- |
| C/C (n=15) | 6 (25.0%) | 9 (34.6%) | 0.638 |
| A/C (n=23) | 11 (45.8%) | 12 (46.2%) |  |
| A/A (n=12) | 7 (29.2%) | 5 (19.2%) |  |
